# Dynamic remodelling of synapses can occur in the absence of the parent cell body

**DOI:** 10.1186/1471-2202-8-79

**Published:** 2007-09-26

**Authors:** Natalia L Bettini, Thomas S Moores, Becki Baxter, Jim Deuchars, Simon H Parson

**Affiliations:** 1 University of Leeds, Institute of Membrane and Systems Biology, Faculty of Biological Sciences, Garstang Building, LS2 9JT, UK; 2 University of Sussex, Sussex Centre for Neuroscience, School of Life Sciences, Falmer, Brighton, BN1 9QG; 3 University of Edinburgh, Section of Anatomy, Centre for Integrative Physiology, Old Medical School, Edinburgh, EH8 9AG, UK

## Abstract

**Background:**

Retraction of nerve terminals is a characteristic feature of development, injury and insult and may herald many neurodegenerative diseases. Although morphological events have been well characterized, we know relatively little about the nature of the underlying cellular machinery. Evidence suggests a strong local component in determining which neuronal branches and synapses are lost, but a greater understanding of this basic neurological process is required. Here we test the hypothesis that nerve terminals are semi-autonomous and able to rapidly respond to local stimuli in the absence of communication with their parent cell body.

**Results:**

We used an isolated preparation consisting of distal peripheral nerve stumps, associated nerve terminals and post-synaptic muscle fibres, maintained in-vitro for up to 3 hrs. In this system synapses are intact but the presynaptic nerve terminal is disconnected from its cell soma. In control preparations synapses were stable for extended periods and did not undergo Wallerian degneration. In contrast, addition of purines triggers rapid changes at synapses. Using fluorescence and electron microscopy we observe ultrastructural and gross morphological events consistent with nerve terminal retraction. We find no evidence of Wallerian or Wallerian-like degeneration in these preparations. Pharmacological experiments implicate pre-synaptic P2X7 receptor subunits as key mediators of these events.

**Conclusion:**

The data presented suggest; first that isolated nerve terminals are able to regulate connectivity independent of signals from the cell body, second that synapses exist in a dynamic state, poised to shift from stability to loss by activating intrinsic mechanisms and molecules, and third that local purines acting at purinergic receptors can trigger these events. A role for ATP receptors in this is not surprising since they are frequently activated during cellular injury, when adenosine tri-phosphate is released from damaged cells. Local control demands that the elements necessary to drive retraction are constitutively present. We hypothesize that pre-existing scaffolds of molecular motors and cytoskeletal proteins could provide the dynamism required to drive such structural changes in nerve terminals in the absence of the cell body.

## Background

Retraction of nerve terminals is a fundamental event in the development and maintenance of the nervous system [1,2], occurring when exuberant neuronal projections are pared back by activity-dependent processes. These events have been extensively studied at the neuromuscular junction (NMJ) during development [3,4], re-innervation [5] and following axotomy [6]. Here, competing nerve terminals at muscle motor endplates are withdrawn bouton-by-bouton in an apparently controlled, piecemeal fashion until the last bouton withers and is re-absorbed into the parent axon [4,7,8]. However, relatively little is known about the cellular mechanisms that govern progressive nerve terminal retraction. This information is particularly pertinent since nerve terminal loss is emerging as a key early event in many neurodegenerative diseases traditionally thought of as disorders of cell bodies [9-12]. In fact nerve terminal loss has now been observed as the earliest event in spinal muscular atrophy [13,14], progressive motor neuropathy [15,16], amyotrophic lateral sclerosis [17], motor neurone degeneration and SOD1 over expression [16]. Additionally, in the CNS Huntington's [18] and Alzheimer's [19] diseases both show early nerve terminal loss.

Available data points to a degree of synaptic independence in the retraction of individual synaptic connections. Observations of single motor units during developmental synapse elimination reveal three quite separate populations of nerve terminals: stable, actively withdrawing and actively enlarging [3,12] and it is difficult to envisage how a single cell soma could directly co-ordinate this range of actions in different parts of its terminal arbour. Adult axons and terminals appear to contain little if any machinery for protein synthesis, but the first signs of regenerative sprouting occurs within a day of axotomy [20,21], which appears too rapid for communication from the site of injury to the cell body and back, even by fast axonal transport. Neonatal synapse elimination can proceed subsequent to axotomy [6] and co-ordinated structural changes at presynaptic nerve terminals (retraction) can occur in the absence of parent cell somas. Axons disconnected from their cell bodies *in vitro *can assemble new growth cones at lesion sites [22-24] and transected axons are able to mount a regenerative response in the absence of cell somas [25]. Taken together these data suggest that the machinery necessary to drive synapse loss (and re-growth) may be constitutively present in the nerve terminal and axon and that communication with the cell body is unnecessary. Although other evidence, primarily from the hippocampus, demonstrates that long-term alterations in synaptic strength at subsets of neurones in a synaptic field may be driven by pre-existing or newly synthesised plasticity related proteins which differentially bind to synapses dependent upon their level of activity. This is thought to be regulated by synaptic tagging [26], where single synapses can show independent behaviour.

In this study we show that nerve terminal retraction can be triggered in the absence of neuronal cell bodies by the extracellular application of an ATP analogue. The nature of the nerve terminal retraction does not resemble Wallerian or Wallerian-like degeneration; rather it resembles 'dying back' neuropathies and synapse elimination.

## Results

### BzATP triggers the rapid loss of nerve terminals in the absence of cell bodies

**Figure 1:**
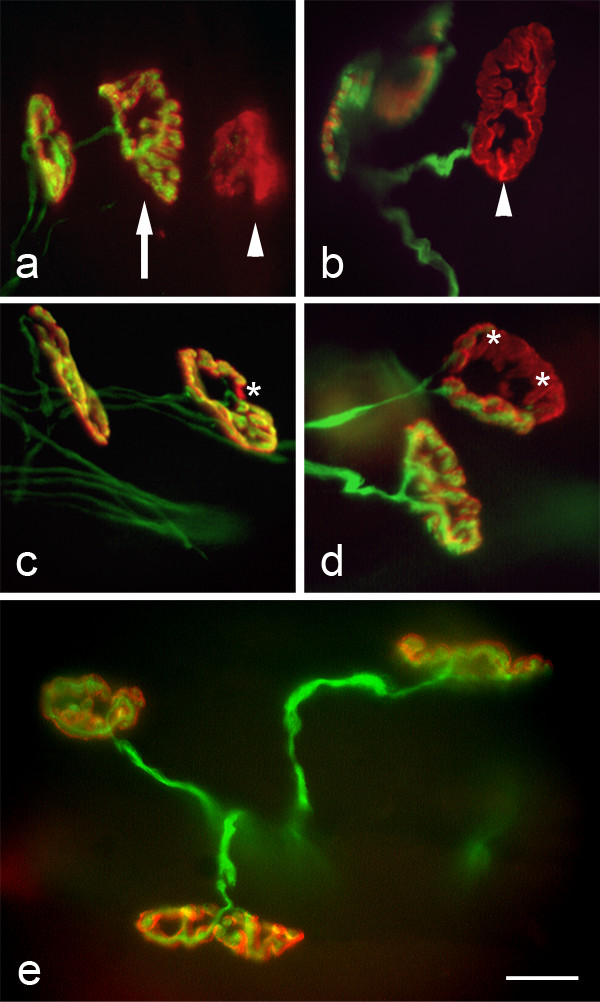
**Nerve terminal bouton loss in response to an extracellular cue occurs in the absence of neuronal cell bodies**. All preparations were fixed in 4% paraformaldehyde prior to immunostaining for neurofilament 165 and SV2 (green: axons and terminals), visualised with a Cy2-conjugated secondary antibody and co-stained with TRITC-αBTX (red: muscle endplates), 165 min after a 15 min BzATP pulse (100 μM). Both 'occupied' (a: arrow) and 'unoccupied' (a: arrowhead) endplates were present, as were unoccupied endplates with intact presynaptic axons (b). Many terminals appeared to be in the process of retraction resulting in minor (c: asterisk) or major (d: asterisks) regions of unoccupied endplates. These were classified as 'intermediate' in subsequent data. Control nerve/muscle preparations, which were maintained in physiological saline for up to 300 min, showed no visible signs of degeneration or retraction of nerve terminals (e). Scale bar a-e = 15 μm

ATP is released following injury, signals local damage [27] and low-sensitivity receptors tuned to respond to such pathophysiological concentrations of ATP are present on motor nerve terminals [28,29]. We therefore tested the effect of activation of ATP receptors at neuromuscular junctions with a relatively stable ATP analogue, BzATP. Preparations consisting of axotomised and isolated peripheral nerve and skeletal muscle were dissected and placed into oxygenated physiological saline, after a brief (15 min) pulse of BzATP (100 μM) they were returned to the oxygenated saline solution for 165 min. Preparations were then fixed and immunostained for components of nerve terminals (neurofilament and synaptic vesicles) and muscle endplates (acetylcholine receptors). BzATP-treated preparations demonstrated a variety of morphologies; typically endplates were either fully occupied by intact nerve terminals (Occupied: Fig 1a, arrow) or devoid of any innervating nerve terminal (Unoccupied: Fig 1a, b, arrowheads). Intact presynaptic axons were often seen immediately adjacent to unoccupied endplates (Fig 1b). Other terminals appeared to lie between these two extremes and had lost either a minority (Fig 1c, asterisk) or a majority (Fig 1d, asterisks) of nerve terminal boutons (Intermediate: Fig 1b–d). Remaining nerve terminals and nerve terminal boutons appeared healthy, maintained connectivity with the intramuscular nerve and were indistinguishable from those seen in control (300 min physiological saline) preparations (Fig 1e).

**Figure 2:**
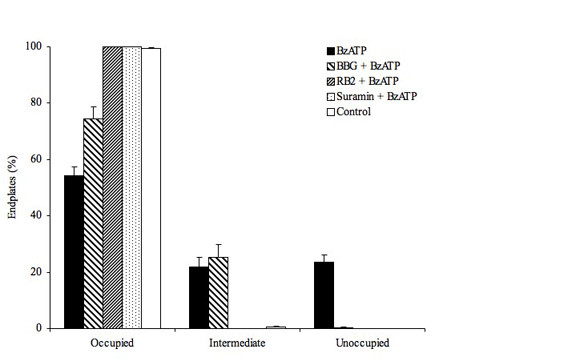
**Nerve terminal loss appears to be triggered via activation of purinoceptors**. Figure showing the proportion of occupied, intermediate and unoccupied muscle endplates present in: 300 min control (open bar); 165 min subsequent to a 15 min BzATP pulse (100 μM: filled bar); 165 min subsequent to a 15 min BzATP pulse (filled bar) in the presence of Brilliant Blue G (a selective P2X7 receptor antagonist: BBG, 1 μM: broad diagonal cross-hatching); 165 min subsequent to a 15 min BzATP pulse in the presence of Reactive Blue 2 (which blocks P2Y and P2X receptor subunits: 100 μM, RB2: narrow diagonal cross-hatching) or 165 min subsequent to a 15 min BzATP pulse in the presence of Suramin (a broad spectrum P2 receptor agonist: 100 μM: stipple). All data is mean ± SEM of multiple repeats (see text for n values).

Quantitative data taken from the preparations described above are shown in figure 2. In summary, data for experimental (BzATP-treated, solid bars) preparations were as follows: occupied, 54 ± 3%; intermediate, 22 ± 3%; unoccupied, 24 ± 2%, N = 4, n = 350. These data demonstrate considerable nerve terminal loss, with almost half of the neuromuscular junctions showing characteristics consistent with retraction. By comparison control preparations (open bars), which were maintained in physiological saline for 180 min had fully occupied muscle endplates (Occupied, 100%, N = 6, n = 550) and were indistinguishable from preparations fixed immediately after dissection (Occupied, 100%, N = 3, n = 150). The reduction in fully occupied muscle endplates triggered by the addition of BzATP was significantly different from control preparations (P < 0.001, Kruskal-Wallis with Dunn's post hoc multiple comparison test).

**Figure 3:**
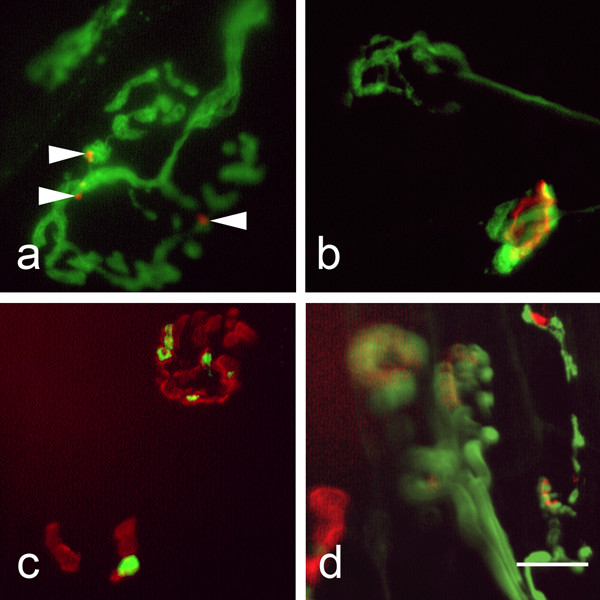
**Some nerve terminals and muscle endplates fragmented in response to BzATP**. All preparations were fixed in 4% paraformaldehyde prior to immunostaining for neurofilament 165 and SV2 (green: axons and terminals), visualised with a Cy2-conjugated secondary antibody and co-stained with TRITC-αBTX (red: muscle endplates), 165 min after a 15 min BzATP (100 μM) pulse. Nerve terminals associated with either fragmented (a), or absent (b) muscle endplates were seen, as were fragmented nerve terminals contacting either apparently normal (c), or fragmented endplates (c, d). Scale bar a, d = 15 μm, b, c = 30 μm

During the course of these experiments we occasionally found intact nerve terminals associated with fragmented endplates (Fig 3a, arrowheads), or lying above muscle fibres where no muscle endplate was present (Fig 3b). We also found several examples of nerve terminals which appeared to be in the process of fragmentation, either in isolation (Fig 3c upper) or possibly in conjunction with endplates (Fig 3c lower, Fig 3d). Similar appearances were not seen in control preparations. These junctions constituted less than 1% of the total and were classified as intermediate for the purposes of quantification.

### BzATP acts at presynaptic purinoceptors to trigger nerve terminal loss

ATP acts at a range of purinoceptors, though our previous studies have only been able to demonstrate P2X7 receptor subunits (P2X7RS) on motor nerve terminals [29]. However, there is evidence for the presence of P2Y receptor subunits on Schwann cells [30] and muscle fibres [31]. We therefore further investigated the possible involvement of purinoceptors in the BzATP-induced response with selective pharmacological agents. Pre-incubation with a selective antagonist for P2X7RS, Brilliant blue G (BBG: 1 μM) either partially blocked or slowed BzATP-induced nerve terminal loss (occupied, 74 ± 4%; intermediate, 25 ± 5%; unoccupied, 0%, N = 3, n = 300). Reactive Blue 2 (100 μM), which is reported to block P2Y and P2X7 receptor subunits, completely blocked BzATP-induced nerve terminal retraction (occupied, 100%, N = 3, n = 300). Complete block of BzATP-induced retraction was also obtained with the broad-spectrum P2 antagonist Suramin (100 μM: occupied, 100%, N = 2, n = 200). Quantitative data from these experiments are summarized in Figure 2.

### Remaining axons and nerve terminals transmit action potentials and recycle neurotransmitter

**Figure 4:**
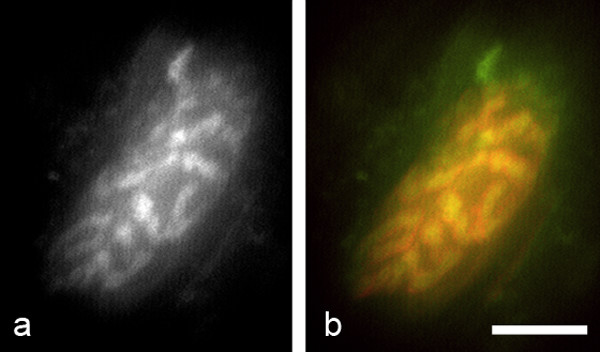
**Intact nerve terminals were capable of releasing and recycling synaptic vesicles subsequent to the addition of BzATP**. The fluorescent vital styryl dye RH414 was used to label recycling synaptic vesicles by indirect nerve stimulation at the end of the 180 min experimental period and just prior to fixation. Remaining nerve terminals were brightly labelled with the dye (a) and fully occupied underlying motor endplates (b). A grayscale image of a single nerve terminal is shown in panel a, while the same terminal (coloured green) is seen to fully occupy a singe motor endplate (coloured red) in the composite image. Scale bar = 10 μm

The failure to propagate compound action potentials [32] and to recycle neurotransmitter are amongst the first signs of neuronal degeneration. We therefore used the vital fluorescent dye RH414 to label nerve terminals 150 min subsequent to a 30 min exposure to BzATP (100 μM). Loading is carried out by electrical stimulation of the intramuscular nerve, which generates nerve action potentials, which then propagate into the nerve terminal and trigger vesicle release. Imaging was carried out 60 min after dye loading to allow for washout of unbound dye (240 min in total). We were able to label synaptic vesicles in motor nerve terminals (Fig 4), which is an indirect measure of their physiological integrity.

### Early ultrastructural events at neuromuscular junctions are consistent with nerve terminal retraction

**Figure 5:**
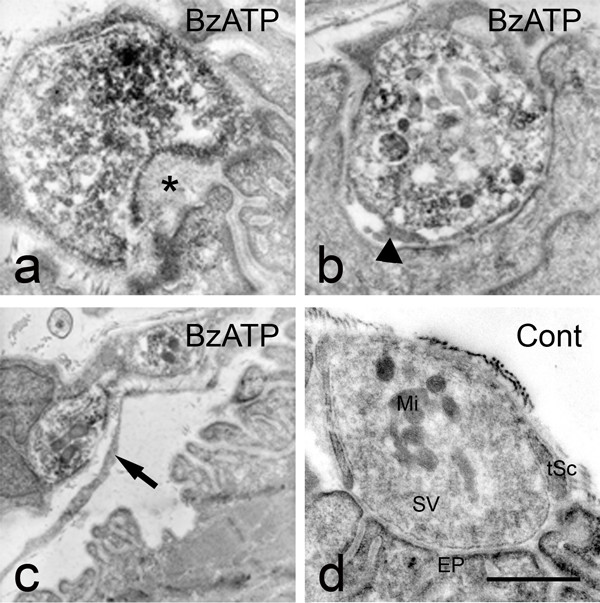
**Ultrastructural events indicate that nerve terminal loss progresses by retraction**. Nerve and muscle preparations were fixed for immuno-electron microscopy and labelled for P2X7 receptor subunits (dark DAB reaction product) to facilitate localisation. When preparations were fixed and immunostained subsequent to a 30 min BzATP (100 μM) pulse frequent abnormal appearances were found. These included; widened synaptic clefts with the appearance of mega-omega profiles (asterisk) (a), fine processes of terminal Schwann cells invaded the synaptic cleft (b: arrowhead), boutons which were detached from the muscle endplate and where terminal Schwann cell processes had come to intervene between the two (c: arrow). In control nerve muscle preparations incubated in physiological saline for 30 min prior to fixation and immunostaining (d), motor nerve terminal boutons contained reaction product for P2X7RS, intact mitochondria (Mi), densely packed synaptic vesicles (SV), were closely apposed to muscle endplates (EP) and overlain by terminal Schwann cell (tSc). a, b, d = 500 nM, c = 1 μM

To further investigate ultrastructural events during the rapid-onset retraction events observed following application of BzATP we carried out immuno-electron microscopy. Isolated peripheral nerve and skeletal muscle preparations were incubated in BzATP (100 μM) for 30 min, immediately fixed and immunostained for P2X7 receptors. Nerve terminal boutons were immunopositive for P2X7 receptors and exhibited evidence consistent with retraction from post-synaptic sites. This included: dilation of synaptic clefts and appearance of mega-omega profiles in the pre-junctional, pre-synaptic membrane (asterisk, Fig 5a); presence of small cellular profiles intervening between pre and post synaptic membranes within the synaptic cleft (Fig 5b, arrowhead); detachment of terminal boutons from the postsynaptic membrane and insertion of terminal Schwann cell process between pre and postsynaptic elements (arrow, Fig 5c). In each case there was no evidence of alteration to the post-synaptic membrane. The synaptic gutters appeared unaltered and widening of the synaptic cleft appeared to be entirely due to movement of the presynaptic nerve terminal away from the surface of the post-synaptic muscle fibre membrane. The ultrastructural changes observed were not consistent with Wallerian degeneration, as axons were intact and withdrawing nerve terminal boutons contained intact mitochondria and were not vacuolated [33-35]. Control preparations that were fixed after 30 min incubation in physiological saline displayed normal morphology including: narrow synaptic clefts, intact mitochondria (Mi), densely packed synaptic vesicles (SV) and overlying terminal Schwann cells (TSc, Fig. 5d). These studies clearly indicate that ultrastructural events consistent with retraction of nerve terminal boutons begin as little as 30 minutes after the addition of BzATP.

**Figure 6:**
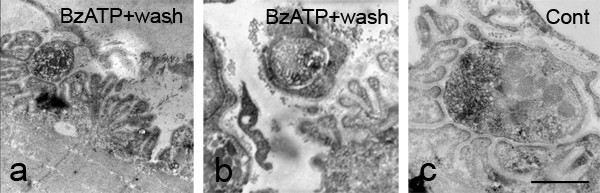
**Ultrastructural changes compatible with nerve terminal retraction continue after removal of BzATP**. When nerve/muscle preparations were exposed to a 30 min BzATP (100 μM) pulse followed by a 60 min washout period prior to fixation, many nerve terminal boutons were in advanced states of retraction. This was manifested as vacant regions of muscle endplate (a) and vesicle-filled boutons that were clearly wrapped by Schwann cell processes (b). Control preparations maintained in physiological saline for 90 min (c) were identical in appearance to freshly fixed preparations (compare with figure 5a). Scale bar a, b = 1 μM, c = 500 nM

To further investigate events subsequent to BzATP incubation, we introduced a 60 min drug washout period after the 30 min BzATP incubation, but prior to fixation. These showed further evidence of ongoing nerve terminal loss including vacant areas of muscle endplate with no overlying nerve terminal bouton (Fig 6a, arrows) and retracting boutons wrapped in Schwann cell processes (Fig 6b). The presence of intact mitochondria and absence of cytoplasmic vacuolation in neurones, coupled with the presence of characteristic striations in muscle (Fig 6a), suggested that neither had degenerated during the course of the extended experimental window. Maintaining untreated (control) preparations for this extended period (90 min) prior to fixation did not result in any obvious changes in gross or fine structure (Fig 6c).

## Discussion

In this study we demonstrate that in preparations where distal nerve axons, terminals and associated skeletal muscle fibres, BzATP can trigger the retraction of nerve terminals from post-synaptic muscle fibre endplates even though they are no longer connected to their parent cell bodies. The events triggered most closely resemble retraction events during neuromuscular synapse elimination. This action of BzATP appears to be mediated by presynaptic P2X7 receptors.

### Isolated axons and terminals can undergo dynamic cellular re-arrangements

In these experiments we show that a brief pharmacological signal can trigger rapid and significant morphological events in cell fragments consisting of isolated nerve terminals and attached distal axon stumps. We suggest that the morphology of these events most closely resembles nerve terminal retraction during developmental synapse elimination [4,6,33,36], following nerve section in the slow Wallerian degeneration mouse [6] and in motor neurone disease [17]. We also observed fragmentation of pre and/or postsynaptic sites at a small number of neuromuscular junctions and interestingly similar synaptic responses have also been observed during developmental synapse elimination [37] and in mouse models of spinal muscular atrophy [14]. Therefore we suggest that the events seen here are part of the normal physiological/pathophysiological repertoire of neurones, which have been triggered by activation of pre-synaptic purinergic receptors. It will be interesting to determine the exact rate and timecourse of the events described here by using confocal and timelapse imaging. However, this may not in fact further illuminate the underlying molecules and mechanisms at play. Significantly, during the course of these studies we found no evidence of gross, injury-induced degenerative events up to and including 5 hr after axons were cut during dissection. This agrees with our previous findings which indicated that Wallerian degeneration does not begin until at least 12 hours after dissection [6]. Specifically, we saw no evidence of terminal Schwann cells engulfing degenerating nerve terminal boutons in-situ, mitochondrial degradation or cytoplasmic vacuolation [32,38], which would indicate degenerative events. We did however, see partially occupied 'intermediate' junctions (Fig 1c, d) and intact nerve terminal boutons which had apparently withdrawn from post-synaptic sites (Fig 5c), both of which are atypical of degenerative changes, and characteristic of retraction events. Occasionally we found evidence of fragmented or absent muscle endplates, associated with intact or fragmented nerve terminals. As P2X2 receptor subunits have recently been shown to have a role in the formation of neuromuscular junctions [39], it is possible that this indicates a direct effect on post-synaptic structures or nerve to muscle communication pathways.

Several other groups have described dramatic morphological events at the neuromuscular junction subsequent to insult. Is it likely then that the events described here are simply a response to toxic insult? Addition of a component of Black Widow Spider venom, α-latrotoxin [40], triggers massive exocytosis of vesicles which produce presynaptic 'arches', similar in appearance to the mega-omega profiles described here. However, these junctions recover and do not progress to disconnection of nerve terminals from muscle fibre membranes or insertion of Schwann cell membranes into synaptic clefts. Blocking of axoplasmic flow by the addition of colchicine [41] after a period of days leads to an increased density of synaptic vesicles, mitochondrial disruption, retraction and Schwann cell wrapping of nerve terminal boutons. However, we did not see mitochondrial damage or obvious signs of synaptic vesicle accumulation, if anything vesicles were slightly depleted compared to control preparations. In addition, colchicine produces gross changes in the post-synaptic muscle fibre membrane associated with the loss of junctional folds as the nerve terminal withdraws. Complement-mediated anti-ganglioside attack [42] produces electron lucent nerve terminals with damaged mitochondria, invasion of Schwann cells into the synaptic cleft and wrapping of nerve terminal boutons. We did not see evidence of such degenerative changes, though we did observe similar insertion and wrapping of nerve terminal boutons by Schwann cell processes, but this is also a common feature of synapse elimination [33,36,37]. All of these situations have disruption of mitochondria as a significant, and often early event, which we did not observe following treatment with BzATP. Further, nerve terminals exhibited selective vulnerability to the BzATP pulse, which is not the case in toxic injury. We would predict that similar events could be demonstrated in vivo and on non-axotomised nerve terminals [6], and this is an avenue which will be pursued in future studies.

### Activation of P2 receptors trigger nerve terminal loss

We show that a stable analogue of ATP triggers structural changes in nerve terminals. BzATP is relatively selective for P2X7RS, but more importantly this is so far the only member of the ionotropic family of P2X receptors reported on motor nerve terminals [28,29]. P2X receptors have however, been described on muscle fibres [39] and Schwann cells [43]. The validity of tools used to demonstrate P2X7RS on neurones have been questioned, but the current consensus is that the original reports [28] are accurate [44]. We suggest that BzATP acts, at least in part, on P2X7RS. BzATP-induced nerve terminal retraction was incompletely prevented by the P2X7RS selective blocker BBG, but completely prevented by RB2 and Suramin, which block both P2X and P2Y receptor, subtypes. Therefore it is likely that BzATP also targets P2Y receptors in these experiments. We do not know to what extent P2X and P2Y receptors work via common or separate signalling pathways and currently available drugs are not sufficiently selective to allow us to unravel the relative involvement of these sub-families at the present time. Interestingly, P2Y receptors have been reported on both Schwann cells [45] and muscle fibres [46], which could provide potential pathways for the observations of Schwann cell invasion and endplate dismantling. Activation of P2X7RS can open a cation-permeable ionotropic channel or a large (membrane permeating) pore [47]. We have previously demonstrated that BzATP does not open a large pore at motor nerve terminals by simultaneous incubation in a solution of the membrane-impermeant fluorochrome, 6-carboxyfluoroscein (see figure 5 in [29]). This suggests that if activation of P2X7RS is a key event in this response, it is the ionotropic channel and not the membrane permeabilising large pore that is responsible.

When might ATP rise to sufficient levels to trigger these responses in vivo? First, following direct muscle trauma when loss of membrane integrity leads to the release of muscle sarcoplasm containing approximately 8 mM ATP [48] into the extracellular space. Second, during tissue ischaemia when both neurones and muscle fibres release ATP [49,50] and ionic gradients across cellular membranes collapse resulting in a fall in the concentrations of extracellular divalent cations [51]. This combination of factors facilitates P2X7RS activation by removal of a resting cation-block [43,52]. One other group have described what appear to be similar retraction events following ischaemia in a preliminary report [53], and another have implicated P2X7RS in hypoxic damage at synapses in the CNS [54], hinting that ATP could be a key factor in hypoxic damage. It is interesting to note that P2 receptor antagonists improve recovery after spinal cord injury [55], pointing to a possible general mechanism for dynamic rearrangement of synapses through nerve terminal loss following chronic or acute injury.

### Autonomous events in nerve terminals

We have demonstrated that BzATP triggers a controlled retraction of nerve terminals from post-synaptic sites. Importantly, this occurs in isolated nerve-muscle preparations where all connectivity with the parent motor neurone cell bodies has been abolished. The heterogeneity of synaptic responses recorded is similar to events described in single motor units during developmental synapse elimination [4,35] and following axotomy in mutant slow Wallerian degenerating (Wld^s^) mice [6]. These observations, taken together with other data [4,6], support the notion that nerve terminals are autonomous or 'compartmentalised' [56], each compartment containing specific elements/processes enabling them to respond in different ways to environmental signals. If local, compartmentalised processes are important in retraction events, how are they controlled? Changes in cell shape require alterations to the cytoskeleton and it seems unlikely that new cytoskeletal elements could be generated and/or assembled within the time frame of these experiments, especially in the absence of neuronal cell bodies. An attractive hypothesis therefore is that sufficient cytoskeletal elements to drive nerve terminal retraction are constitutively present in distal axons and terminals and that re-arrangement of these drive retraction events. Non-muscle myosins IIA and IIB drive neurite elongation and retraction in culture [57,58] and we have previously shown that these are present in motor nerve terminals [59]. Here a pre-assembled 'molecular clutch' [60] regulated by the differential activation of opposing myosin motors by kinases could drive cytoskeletal rearrangement, and therefore morphological change, without the requirement for generation of new cytoskeletal elements. It remains unclear how purinergic receptors might be linked to the cytoskeleton, but evidence exists to link similar pathways in other systems. Extracellular purines acting on membrane-bound receptors trigger dissagregation of the actin cytoskeleton in the WRK-1 (mouse mammary tumour) cell-line. This may be related to differential activation of molecular motors [61]. Proteomic analysis of the P2X7R indicates several cytoskeletal elements present within the receptor complex [62] and one of these, supervillin, binds to actin and non-muscle myosin II. Supervillin is an important adapter protein in the organisation of attachments of the cytoskeleton to dynamic regions of membranes [63] which could drive shape changes at nerve terminals.

## Conclusion

In summary, we provide evidence that following disconnection from their parent cell bodies, activation of P2X7 receptors on nerve terminals by BzATP triggers synaptic retraction. This retraction closely resembles similar events occurring during synapse elimination and motor neurone disease. We suggest that molecules and mechanisms constitutively present in the distal axon and terminal are sufficient to drive this retraction response. Further, we speculate that signals from the parent cell soma may not be necessary to control retraction during synaptic plasticity.

## Methods

### Fluorescence microscopy

Adult C57BL/6 mice were killed by CO_2 _intoxication, and intact preparations of lumbrical muscles, plantaris tendon and sciatic/tibial nerves were dissected and pinned out at resting length in Sylgard-lined Petri dishes containing oxygenated (95%O_2_/5%CO_2_), calcium and magnesium free physiological saline (122.4 mM NaCl, 5 mM KCl, 0.4 mM NaH_2_PO_4_, 23.8 mM NaHCO_3_, 5.6 mM Glucose, 5.5 mM Hepes, pH 7.2–7.4) at room temperature. These were transferred to oxygenated physiological saline as above, and any muscles which failed to twitch in response to nerve stimulation were excluded from further assay. Preparations were either maintained throughout in oxygenated physiological saline or alternatively in the same saline supplemented with several different purinergic agonist and antagonists. Purine receptor agonists and antagonists used were as follows: 2'-3'-O-(4-benzoylbenzoyl)-adenosine 5'-triphosphate (BzATP: 100 μM), Brilliant blue G (BBG: 1 μM), Reactive Blue 2 (RB2: 100 μM), Suramin (100 μM). All experiments began within 60 min of animal sacrifice.

After the experimental period (3 hrs), preparations were fixed in 4% paraformaldehyde for 30 mins and stained for immunofluorescence microscopy. Synaptic vesicles and neurofilaments were labelled with a combination of SV2 (1:500) and NF165 (1:250, developed by T.M. Jessel and J. Dodd, and obtained from the Developmental Studies Hybridoma Bank developed under the auspices of NICHD and maintained by the University of Iowa, Department of Biological Sciences, Iowa City, USA), and visualised with Cy2- (1:200, donkey anti mouse: Jackson laboratories, USA) secondary antibody. Post-synaptic acetylcholine receptors were labelled with tetramethyl rhodamine isothiocyanate conjugated ãbungarotoxin (5 μg/ml, TRITC-a-BTX: Molecular Probes, Leiden). Slides were coverslipped in 2.5% n-propyl gallate in glycerol and viewed with epifluorescence illumination. In preliminary experiments we determined that a 15 min pulse of BzATP was the optimum trigger for nerve terminal retraction.

### Electron microscopy

Mice were killed and handled as above, but in this case intact preparations of flexor digitorum brevis muscles and associated sciatic/tibial nerve were dissected and placed Immediately after dissection into freshly prepared and oxygenated physiological saline. Addition of chemicals began within 60 minutes of animal sacrifice. BzATP (100 μM) was added for 30 min and preparations were fixed in buffered 4% paraformaldehyde/0.2% glutaraldehyde, pH 7.2 either immediately or subsequent to a 60 min washout period. Muscles were fixed and labelled for electron microscopic localisation of P2X7 receptor subunits (P2X7RS) as previously described [29].

### Vital imaging

Preparations were treated identically to those above, except at the end of the 3 hr period, they were vitally labeled and imaged. Preparations were loaded with the vital styryl dye RH414 (10 μg/ml; Molecular Probes) in physiological saline solution by nerve stimulation (suprathreshold 0.1 msec pulse trains delivered at 10 Hz for 10 min to the intercostal nerve via asuction electrode), followed by at least 1 hr washing in oxygenated physiological saline. Preparations were transferred to the microscope stage and terminals were imaged.

### Quantification

25 sequentially identified endplates from randomly oriented immunostained lumbrical muscles were identified and quantified for the degree of congruence between overlying nerve terminal and muscle endplate from four muscles per hindfoot. Three categories were used; 'occupied', denoting complete occupancy; 'intermediate', where either endplate was partially occupied by nerve terminal or was fragmented; 'unoccupied', where no nerve terminal was present at the endplate. Occasionally, damaged muscle fibres or unusually deep endplates were difficult to accurately assess, and were discarded from the data set. Data are presented as mean ± SEM, N = number of mice, n = number of endplates. GraphPad Prism (Graphpad software) was used to carry out statistical tests.

## Authors' contributions

NLB carried out and analysed the fluorescence microscopy data and drafted the manuscript, TSM carried out the electron microscopy, BB carried out and analysed additional fluorescence microscopy, JD participated in experimental design and helped draft the manuscript, SHP carried out preliminary experiments, conceived the study and drafted the manuscript.
